# The Impact of THC and CBD in Schizophrenia: A Systematic Review

**DOI:** 10.3389/fpsyt.2021.694394

**Published:** 2021-07-23

**Authors:** Saeed Ahmed, Robert M. Roth, Corneliu N. Stanciu, Mary F. Brunette

**Affiliations:** ^1^Department of Psychiatry, Rutland Regional Medical Center, Rutland, VT, United States; ^2^Vermont Hub-and-Spoke System of Care, West Ridge Center at Rutland Regional Medical Center, Rutland, VT, United States; ^3^New Hampshire Hospital, Concord, NH, United States; ^4^Department of Psychiatry, Dartmouth-Hitchcock Medical Center, Lebanon, NH, United States; ^5^Geisel School of Medicine, Dartmouth College, Hanover, NH, United States; ^6^Bureau of Mental Health Services, Concord, NH, United States

**Keywords:** cannabis, marijuana, Schizophrenia, psychosis, CBD, THC, legalization, fMRI

## Abstract

**Background:** People with schizophrenia are more likely to develop cannabis use disorder (CUD) and experience worse outcomes with use. Yet as cannabis is legalized for medical and recreational use, there is interest in its therapeutic potential.

**Objectives:** To conduct a systematic review summarizing the design and results of controlled trials using defined doses of THC and CBD in schizophrenia.

**Method:** A keyword search of eight online literature databases identified 11 eligible reports.

**Results:** One placebo controlled trial (13 stable patients without CUD) found that intravenous THC increased psychosis and worsened learning/recall. Two reports of a functional magnetic resonance (fMRI) study of smoked or oral THC in 12 abstinent patients with schizophrenia and CUD found no change in symptoms and cognition, and an amelioration of impaired resting state brain function in areas implicated in reward function and the default mode network. One 4 week trial in acutely psychotic inpatients without CUD (mean age 30 y) found 800 mg CBD to be similarly efficacious to amisupride in improving psychosis and cognition. Two 6 week studies of CBD augmentation of antipsychotics in stable outpatients reported mixed results: CBD 600 mg was not more effective than placebo; CBD 1,000 mg reduced symptoms in a sample that did not exclude cannabis use and CUD. A brain fMRI and proton magnetic resonance spectroscopy study of single dose CBD in a sample that did not exclude CUD and cannabis use found that CBD improved symptoms and brain function during a learning/recall task and was associated with increased hippocampal glutamate.

**Discussion:** There is substantial heterogeneity across studies in dose, method of drug delivery, length of treatment, patient age, whether patients with cannabis use/CUD were included or excluded, and whether patients were using antipsychotic medication.

**Conclusion:** There is insufficient evidence for an effect of THC or CBD on symptoms, cognition, and neuroimaging measures of brain function in schizophrenia. At this time, research does not support recommending medical cannabis (THC or CBD) for treating patients with schizophrenia. Further research should examine THC and CBD in schizophrenia with and without comorbid CUD and consider the role of CBD in mitigating symptom exacerbation from THC.

## Introduction

Schizophrenia is a chronic neurodevelopmental disorder experienced by 0.5 to 1.0% of the population worldwide (1, 2). This condition typically begins in late adolescence or early adulthood and includes positive symptoms, such as hallucinations, and negative symptoms, such as avolition. Cognitive impairments, such as with attention and working memory, are core features of schizophrenia, and an impaired ability to anticipate reward has also been documented (3). Significant anxiety is common, though not a core symptom of schizophrenia (4, 5). Co-occurring substance use disorders are more common in people with schizophrenia than the general population, and cannabis is the most common illicit drug used by people with this condition (6–9). Up to 43% of people with schizophrenia develop a cannabis use disorder (CUD) (10–13) compared to 6.3% in the general population (14).

Interestingly, epidemiological studies have demonstrated that heavy cannabis use in early adolescence is associated with an increased risk for the development of new psychotic symptoms and schizophrenia-spectrum disorders (15–19). A dose-response relationship has been observed, with a higher incidence of schizophrenia found in heavy cannabis users compared to light or non-users (17). Additionally, among people who have an established schizophrenia spectrum disorder, observational studies have shown that recreational use of cannabis and cannabis use disorder are associated with worse symptoms and course of illness (20–23). As we will further delineate below, examining the effects of both THC and CBD, alone and together, may help the field better understand the mechanism of action of the effects of cannabis, the pathophysiology of schizophrenia, and whether there is any therapeutic role for these cannabis components in people with schizophrenia both with and without cannabis use disorders.

Cannabis is a genus of plants with several species containing over 100 types of cannabinoids. Species are bred to promote varying levels of cannabinoids, especially (–)-*trans*-Δ^9^-tetrahydrocannabinol (THC) and cannabidiol (CBD), which have differing effects. THC is responsible for the intoxicating “high” of cannabis and is likely a component of cannabis that is responsible for the development of CUD in about 10% of users [for review, see (24)]. In contrast, CBD does not appear to cause intoxication, nor is it reinforcing (25, 26).

Controlled laboratory studies in healthy participants have demonstrated that THC administration results in acute psychotic symptoms and transient dose-related cognitive impairments, including in working memory and the executive control of attention, in up to 50% of healthy individuals (27), and for review, see (28). Some studies show a dose effect for psychosis [e.g., (27)]. Pre-treatment with CBD has been shown to mitigate such THC-induced symptoms and impairments (29–32), but not the positive and reinforcing effects (26). Notably, the THC content in typical street cannabis has risen from ~4% in 1995 to ~12% in 2014 (33), and the proportion of CBD to THC has diminished to almost zero in many strains, although high CBD strains are also available (34). THC and CBD are used to create a variety of high potency products for sale especially in locales where medicinal and recreational cannabis is legal. Thus, easily available high-THC recreational cannabis has strong potential to cause negative effects.

Although use of recreational cannabis (assumed to be high in THC and low in CBD) has been associated with worse outcomes in schizophrenia, several case reports suggested that CBD itself might be beneficial in the treatment of psychosis (35, 36). A more recent cross-sectional report indicated that use of cannabis with high CBD content was associated with significantly lower psychotic symptoms in patients with schizophrenia (32). Research using animal models examining CBD's anti-psychotic-like properties determined that CBD leads to behavioral responses similar to responses to an atypical antipsychotic drug (35), contributing to interest in testing CBD for its ability to improve symptoms in patients with schizophrenia.

As Canada and parts of the U.S. have legalized cannabis for recreational (16 states as of 2021) or medical (12 states as of 2021) purposes (37), the production and sales of cannabis have skyrocketed and the public increasingly perceives cannabis as helpful rather than harmful. Recent surveys showed that almost half of Americans indicated they believed that cannabis may provide relief from anxiety and depression (38). Thus, in locales where cannabis is legal for recreational or medical use, many people seek cannabis to address mental health issues. For example, in one U.S. report, over a third of people who used medical cannabis reported using it to reduce anxiety (39), and several Canadian studies reported that cannabis was widely used to treat anxiety, depression, and sleep (40, 41), symptoms common across an array of mental health conditions, including psychotic disorders (42).

Thus, as stakeholders are increasingly interested in the possible therapeutic effects of cannabis, they need reliable information about the effects of THC and CBD, particularly among vulnerable populations such as people with schizophrenia. Several prior reviews have addressed the effects of THC or CBD in people with schizophrenia (43–50). We sought to provide an updated review, as well as detailed and critical review of the literature including studies of both CBD and THC considered together, as well as a critical review of the research methods, quality of the research, and directness of evidence for each study (51), focusing on randomized controlled trials (RCTs), as they provide the highest level of evidence. This review therefore provides a review of the evidence of the potential benefits and harms of THC and/or CBD in schizophrenia to date. We conducted a systematic review of published prospective, controlled studies testing the impact of THC and/or CBD on symptoms, cognition, and neuroimaging measures of brain function in people with schizophrenia-spectrum disorders.

## Methodology

### Information Source and Search

Literature searches using PubMed/MEDLINE, PsycINFO, PsycARTICLES, CINAHL, EMBASE, Scopus, Cochrane, and Academic One File were conducted for English-language papers published between January 1st, 1970, and June 15, 2021. Search terms included: “cannabidiol AND schizo^*^”; “cannabidiol AND psycho^*^”; “CBD AND schizo^*^”; “CBD AND psycho^*^”; “tetrahydrocannabinol AND schizo^*^”; “tetrahydrocannabinol AND psycho^*^”; “THC AND schizo^*^”; “THC AND psycho^*^.” In addition, we examined recent peer-reviewed scientific reviews of the literature on cannabinoids and psychosis, as well as reference sections of papers garnered from the online literature search, for any other relevant articles.

### Inclusion and Exclusion Criteria

All studies reporting prospective RCTs testing specific doses of whole-plant cannabis, CBD, THC, or both compounds compared with placebo or control condition with standardized assessments of symptoms of psychosis, cognition, and/or neuroimaging in humans with schizophrenia spectrum disorders were considered. Any commercially available or synthetic THC or CBD formulation was accepted, as well as any route of administration for any period of time. Age, sex, and race/ethnicity were not included in the selection criteria. We excluded cross-sectional studies, observational studies without a control condition, studies examining cannabis that did not use a specified dose of THC and/or CBD, CBD used for psychiatric illnesses other than schizophrenia, papers not written in English; studies not reporting original research, and studies with participants less three.

### Assessment of Study Quality

Once studies were selected, we conducted an assessment of study quality using a checklist for the “grading of recommendation, assessment, development, and evaluation (GRADE” approach (51). The GRADE is a widely used, transparent classification system for rating research quality and developing evidence summaries that provides a systematic approach for making clinical practice recommendations (52–54). We used two categories: study quality/risk of bias and directness/indirectness of evidence.

## Results

### Study Selection

**Figure 1:**
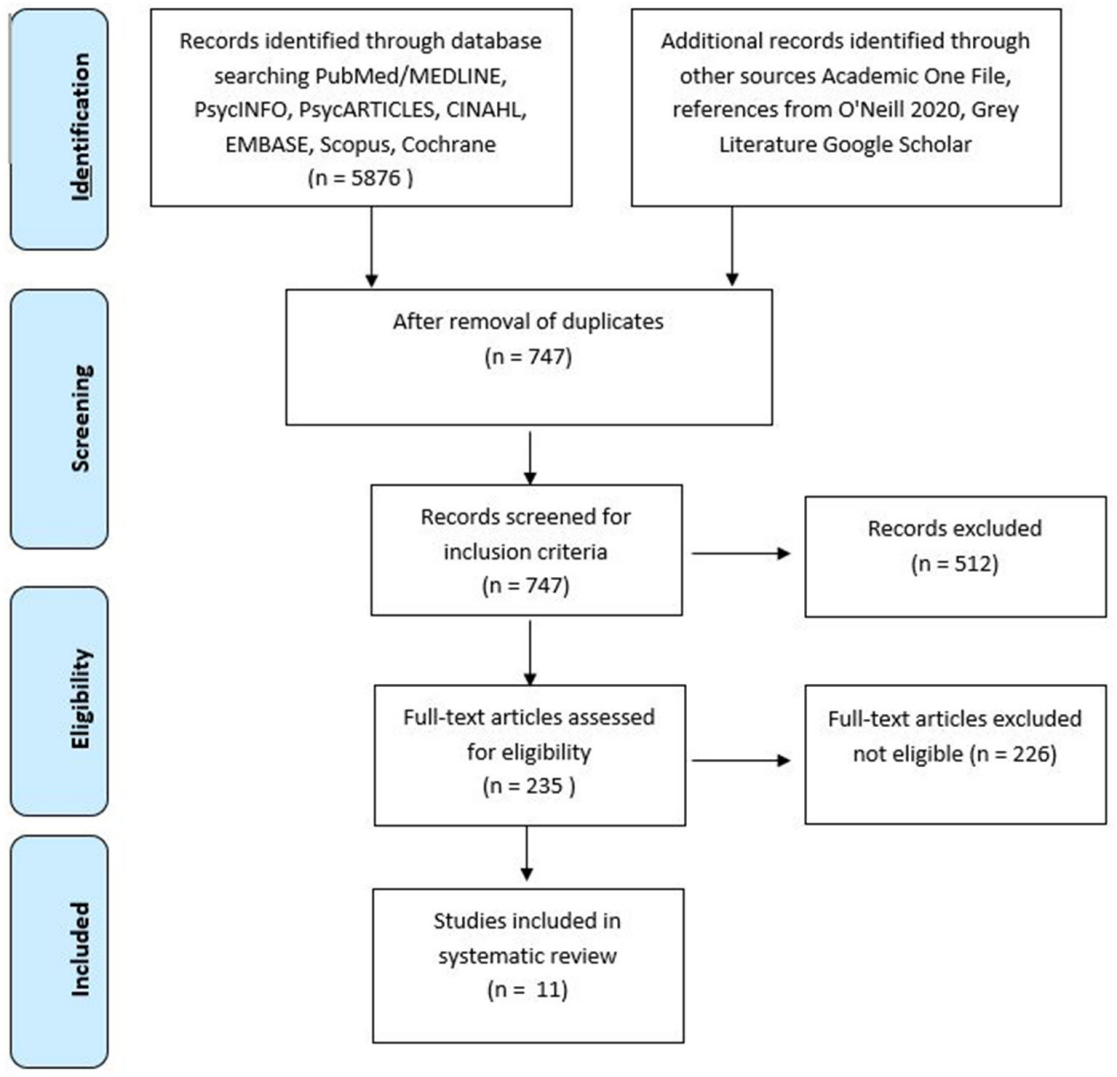
Flow diagram.

The initial search yielded 6,003 reports. After removing duplicates, studies were screened base on titles, resulting in the inclusion of 722 citations. Abstracts were then screened, which resulted in exclusion of 512 citations. The remaining papers (235) were reviewed for eligibility by two authors (C.N.S. and S.A.). Any disagreements were mediated by a third reviewer (MB). A total of 226 papers did not fit the inclusion criteria, resulting in 11 full-text articles that met inclusion criteria. The selection steps are shown in Figure 1.

### Study Characteristics

Table 1 provides the characteristics of the nine prospective, placebo-controlled studies of cannabis, CBD and/or THC. These studies were published between 2005 and 2021 and employed a variety of methods, which are described in the table and below.

**Table 1:** Methods and results of studies of CBD and THC in the treatment of Schizophrenia.

**References**	**Participants**	**Design**	**Substance use criteria**	**Primary outcome measures**	**Findings**	**Symptom scores**
**Studies of THC**						
D'Souza et al. (55)	13 medicated outpatients with SCZ or SCZAF (DSM-IV), mean age 44.46 ± 10.4, 76.9% male. 22 HC, mean age = 29 ± 11.6, 63.6% male	RCT double blind, repeated-measures (at least 1 week apart), within-subject cross-over design of single dose intravenous Δ-9-THC 2.5 mg, 5 mg, or PLB	Excluded Lifetime CUD or recent substance abuse (3 m) or dependence (1 yr), other than nicotine. Abstain from all substances, verified via self-report and urine drug screen	Symptoms: PANSS, CADSS, VAS (high, calm and relaxed, tired, panic)Cognitive: HVLT, Gordon CPT, verbal (letter) fluency testSide effects: BAS, SAS, AIMS	THC worsened: verbal learning and recall; positive symptoms; more prominently for patient group; negative symptoms; clinician- and self-related perceptual alterations THC resulted in a trend toward increased VAS ratings of “panic” and “tired” and rigidity, worse AIMS score and akathisia, and increased plasma prolactin and cortisol	PANSS Total, screening: 34.1 ± 9.4Post THC scores not provided
Fischer et al. (56)	12 medicated outpatients with SCZ and CUD (DSM-IV-TR), mean age [smoked cannabis 36.2 ± 9.6; THC capsule 32.17 ± 8.32, male), 583% male 12 HC, mean age 33.5 ± 7.8, 75% male	RCT double blind, parallel group study of smoked 3.6% THC cannabis cigarette immediately prior to scan (*n =* 6), or 15 mg THC capsule 3 h prior to scan (*n =* 6)Two scan sessions (T1, no drug; T2, drug) at least 1 week apart	Required to have a CUD and recent cannabis use. Excluded other substance use disorders. Abstain from all substances, except nicotine and caffeine >7 days prior to scan verified via TLFB, urine screens, plasma THC	Symptoms: PANSS, VAS (high, liking and craving), CWS, MCQImaging: fMRI resting state functional connectivity of BRC	Reduced connectivity at BL in patients between nucleus accumbens and prefrontal cortical BRC regions (i.e., anterior prefrontal cortex, orbitofrontal cortex, anterior cingulate cortex). Both oral and smoked THC incr connectivity between these regions, which correlated with incr in plasma THC levels	No change after THC (PANSS scores not reported)
Whitfield-Gabrieli et al. (57)	Same as Fischer et al. (56)	Same as Fischer et al. (56)	Same as Fischer et al. (56)	Symptoms: PANSS, VAS (high, liking and craving), CWS, MCQCognition: LNSImaging: fMRI resting state functional connectivity of DMN	At BL, patients had DMN hyperconnectivity that correlated with positive symptoms, and reduced anticorrelation between DMN and ECN. THC reduced DMN hyperconnectivity and increased DMN-ECN anticorrelation. The magnitude of anticorrelation in controls, and in patients after THC, correlated with working memory)	PANSS Positive ScoreBL—T1 (13.82 ± 3.19) orPre-drug—T2 (12.91 ± 3.21)No change after THC (PANSS scores not provided separately from smoked cannabis and oral THC and not reported for T2 after THC)
**Studies of CBD**						
Zuardi et al. (35)	3 unmedicated inpatients with treatment-resistant SCZ (DSM-IV), age 21–22 years, all male	Case Series of 6 week CBD titration up to 1,280 mg/day, PLB lead in and washout, then switch to olanzapine	None reported	Symptoms: BPRS, PANSS-NFunctional: CGISide effects: BAS, SAS, UKU Side effect Rating Scale	CBD 1,280 mg/day associated with: Pt 1—trend toward improved BPRS (general, positive, and negative symptoms); Pt 2—no benefit; Pt 3—“very minimal improvement” of positive and negative symptoms In two patients, symptoms worsened after CBD discontinued. No side effects reported	*BPRS Total*:Patient 1: PLB 19, CBD 10Patient 2; PLB 30, CBD 28Patients 3: PLB 29, CBD 26PANSS-N scores not reported
Hallak et al. (58)	28 outpatients with SCZ (DSM-IV), all BPRS scale scores <2, at least 18 years of age, 64.3% male	Single dose non-randomized, double blind, parallel group study of CBD augmentation 300 mg (*n =* 9) or 600 mg (*n =* 9) or PLB (*n =* 9)	History of substance abuse or adverse reaction to marijuana were excluded	Symptoms: BPRS, PANSSCognition: SCWTOther: electrodermal responsiveness	PLB and 300 mg CBD: less SCWT interference errors during 2nd session, but only a trend for 600 mg CBD group, indicating worse selective attention No group differences in electrodermal responsiveness or symptoms, but no analysis of within-group symptom change reported	*BPRS Total*:PLB: BL 8.6 ± 4.1, drug 7.9 ± 5.76CBD 300 mg: BL 11.3 ± 7, drug 10.9 ± 6;CBD 600 mg: BL 8.9 ± 5.1, drug 8.2 ± 5.9*PANSS Total*:PLB: BL 21.9 ± 6.9, drug 21.9 ± 7.2;CBD 300 mg: BL 23.6 ± 9.4, drug 23.4 ± 9.6CBD 600 mg: BL 20.2 ± 7.7, drug 19.1 ± 7.0
Leweke et al. (59)	42 acutely ill unmedicated inpatients with SCZ (DSM-IV), BPRS Total ≥ 36 and BPRS THOT ≥ 12, 18–50 years of age [CBD mean 29.7 ± 8.3 yr, amisulpride mean 30.6 ± 9.4 yr, 82.1% male	4 week RCT, double blind, parallel group study of CBD augmentation 800 mg (*n =* 20) or amisulpride 800 mg (*n =* 19), 1 week titration and 3 weeks treatment (modified intent-to-treat)	History of SUD or positive urine drug screen (including cannabinoids) were excluded	Symptoms: BPRS, PANSSFunctional: CGISide effects: SAS, EPS	BPRS and PANSS (total, positive, negative, general scores) improved over time in both groups. CBD group had less: extrapyramidal symptoms, weight gain, and prolactin elevation Serum anandamide levels were higher in CBD than amisulpride group, with extent of increase associated with PANSS Total score improvement	PANSS Total ScoresCBD score at BL 91.2 (14.0)Changed-−18.8 (10.7) on day 14, −30.5 (16.4) on day 28Amisulpride score at BL 95.9 (17.1)Changed-−18.8 (19.9) on day 14−30.1 (24.7) day 28
Leweke et al. (60)	Same participants as above 42 acutely ill unmedicated inpatients with SCZ (DSM-IV), BPRS Total ≥ 36 and BPRS THOT ≥ 12, 18–50 years of age [CBD mean 29.7 ± 8.3 yr, amisulpride mean 30.6 ± 9.4 yr, 82.1% Male	Same as above4 week RCT, double blind, parallel group study of CBD augmentation 800 mg (*n =* 20) or amisulpride 800 mg (*n =* 19), 1 week titration and 3 weeks treatment (modified intent-to-treat)	Same as above History of SUD or positive urine drug screen (including cannabinoids)	Symptoms: BPRS, PANSSFunctional: CGICognition: Visual Backward Masking Task, CPT, LNS, SOPT, DRT, AVLT, RCFT, Digit Symbol, TMT, Verbal Fluency Task	From pre- to post-treatment, both groups improved in visual memory, processing speed CBD improved sustained attention and visuomotor coordination Amisulpride improved working memory performance Changes in neurocognitive performance were not systematically associated with symptom improvements nor change in serum anandamide	Differences in cognitive improvement not statistically significant after correction for multiple testsVisual memory (CBD: 0.49, *p =* 0.015 vs. AMI: 0.63, *p* = 0.018); processing speed (CBD: 0.41, *p* = 0.004 vs. AMI: 0.57, *p* = 0.023). Sustained attention (CBD: 0.47, *p =* 0.013 vs. AMI: 0.52, *p =* 0.085); visuomotor coordination (CBD: 0.32, *p =* 0.010 vs. AMI: 0.63, *p =* 0.088). SOPT–AMI: 0.53, *p =* 0.043 vs. CBD: 0.03, *p =* 0.932 and LNS–AMI: 0.67, *p =* 0.017 vs. CBD: 0.08 *p =* 0.755)
Boggs et al. (61)	36 medicated outpatients with SCZ (DSM-IV-TR), 18–65 years of age [CBD mean 48.4 ± 9.3; PLB mean 46.4 ± 9.5], 66.7% to 72.2% male	6 week RCT, double blind, parallel group study of CBD augmentation 300 mg twice daily (*n =* 20) or PLB (*n =* 19)	Diagnosis of substance abuse within 3 months or dependence within 6 months of participation (other than nicotine) were excluded	Symptoms: PANSSCognition: MCCBSide effects: BAS, SAS, AIMS, UKU Side Effect Rating Scale	No difference in reduction in PANSS scores (Total, General, Positive, Negative) over time. PLB but not CBD group had small improvement on MCCB (Composite score, Reasoning and Problem Solving domain scores). CBD group had greater sedation compared to PLB	*PANSS screening visit scores*:*Total*: CBD 76.6 ± 17, PLB 82.7 ± 8.8*Positive*: CBD 18.8 ± 4.7, PLB 20.6 ± 3.8*Negative*: 20.7 ± 4.6, PLB 20.9 ± 4.7*General*: 37.1 ± 10.3, PLB 41.2 ± 5.6
McGuire et al. (62)	88 medicated outpatients with SCZ or related psychotic disorder (DSM-IV), PANSS score <60 at screening excluded, 18–65 years of age (mean 40.8 ± 11.69), 58% male	6 week RCT, double blind, parallel group study of CBD augmentation 500 mg BID (*n =* 43) or PLB (*n =* 45)	Alcohol or substance use history allowed; use of alcohol, cannabis or other substances not prohibited during study; positive baseline urine THC test in 1 CBD and 2 PLB group patients	Symptoms: PANSS, SANSFunctional: GAF, CGI-I, CGI-SCognition: BACSSide effects: SAS	CBD group had greater reduction of positive symptoms and more likely to be rated by treating clinician as having improved and have less severe illness than PCB. CBD showed trend for greater improvement in overall level of functioning, cognition (BACS composite score and executive function domain), and motor speed. No group difference for adverse events or side effects	*PANSS Total*:CBD: BL 79.3 ± 12.5, end of Tx 68.1 ± 14.8PLB: BL 80.6 ± 14.9, end of Tx 71.9 ± 15.5*PANSS Positive*:CBD: BL 18.0 ± 3.9, end of Tx 14.8 ± 4.0; PLB: BL 17.5 ± 3.3, end of Tx 15.7 ± 3.7
O'Neill et al. (63)	15 outpatients (14 medicated) with SZ, SCZAF, or Brief Psychotic Disorder (DSM-IV) within 5 years of diagnosis, mean age 27.73 ± 4.61 years, 66.7% male 19 HC, mean age 23.89 ± 4.15 years, 57.9% male	RCT double blind, repeated-measures (1 week apart), within-subject cross-over design of single dose 600 mg oral CBD or PLB	Allowed: current cannabis abuse, dependence, or use Excluded: Current alcohol or substance dependence; or intoxicated or positive urine drug screen on the day of scanning. No alcohol for 24 h or caffeine for 12 h before sessions. No drugs except cannabis for 2 weeks prior to scan	Symptoms: PANNS, STAI-SImaging: fMRI verbal paired associate learning task completed 3 h after CBD or PLB (13 patients completed both scans)	CBD associated with trend toward reduced median PANSS Total. Compared to HC, patients on PLB had abnormal activation within prefrontal region during verbal encoding, and abnormal prefrontal and mediotemporal activation as well as greater hippocampal-striatal functional connectivity during recall. CBD resulted in partial normalization of activation in these regions, as well as reducing hippocampal-striatal hyperconnectivity	PANSS Total:PLB: T1 48.8 ± 18.9, T3 44.6 ± 18.07CBD: T1 51 ± 20, T3 41.53 ± 11*PANSS Positive*:PLB: T1 12.53 ± 5.62, T3 11.67 ± 4.99CBD: T1 12.93 ± 5.72, T3 10.73 ± 3.41*PANSS Negative*:PLB: T1 12.4 ± 6.4, T3 11.53 ± 6.06CBD: T1 12.47 ± 6.56, T3 10.2 ± 3.05Note: T1 is 60 min pre-drug and T3 270 min post-drug administration
O'Neill et al. (64)	Same participants as above 15 outpatients (14 medicated, 1 non-compliant) with SZ, SCZAF, or Brief Psychotic Disorder (DSM-IV) within 5 years of diagnosis, Mean age 27.73 (4.61, 66.7% male)	Same as aboveDouble-blind, randomized, placebo-controlled, repeated-measures (1 week apart) within-subject cross-over design 600 mg oral CBD or PLB	Same as above Allowed: current cannabis abuse, dependence or use	Symptoms: PANSSImaging: ^1^H-MRS spectra were acquired 180 min after CBD or PLB administration (13 patients completed both scans)	CBD associated with greater improvement in PANSS Total and greater hippocampal glutamate levels compared to PLB (*p =* 0.035). An adjusted multivariable model showed an inverse predictive relationships between hippocampal glutamate and post intervention PANSS (*p =* 0.047), but no relationship to CBD group	PANSS symptom scoresSame as above

*AIMS, Abnormal Involuntary Movements Scale; AMI, amisulpride; AVLT, Auditory-Verbal Learning Test; BACS, Brief Assessment of Cognition in Schizophrenia; BAS, Barnes Akathisia Scale; BPRS, Brief Psychiatric Rating Scale; BPRS THOT, BPRSD Thought Disorder factor; BRC, Brain Reward Circuit; CADSS, Clinician-Administered Dissociative States Scale; CWS, Cannabis Withdrawal Scale; CBD, cannabidiol; CGI, Clinical Global Impressions Scale; CGI-I, Clinical Global Impression-Improvement scale; CGI-S, Clinical Global Impression-Severity scale; CPT, Continuous Performance Test; CUD, cannabis use disorder; DMN, Default Mode Network; ECN, Executive Control Network; EPS, Extrapyramidal Symptom Scale; fMRI, Functional magnetic resonance imaging; GAF, Global Assessment of Functioning; HC, Healthy comparison subjects; LNS, Letter-Number Sequencing test from the Wechsler Adult Intelligence Scale-Third edition; MCCB, MATRICS Consensus Cognitive Battery; MCQ, Marijuana Craving Questionnaire; PANSS, Positive and Negative Syndrome Scale; PLB, placebo; RCT, Randomized Control Trial; RCFT, Rey-Osterrieth Complex Figure Test; SANS, Scale for the Assessment of Negative Symptoms; SAS, Simpson Angus Scale; SCZ, Schizophrenia; SCZAF, schizoaffective disorder; SCWT, Stroop Color Word Test; SOPT, Self-Ordered Pointing Task; STAI-S, State Trait Anxiety Inventory state subscale; SUD, substance use disorder; THC, Tetrahydrocannabinol; TLFB, Timeline Follow-Back; TMT, Trail Making Test*.

#### Characteristics of CBD Studies

Four RCTs (reported in six papers) met inclusion/exclusion criteria. Three were longitudinal treatment studies that were 4 to 6 weeks in duration (59, 61, 62), and one was a single dose laboratory study reported in two papers that used functional magnetic resonance imaging (fMRI) (63) and proton magnetic resonance spectroscopy (^1^H-MRS) (64). We also included one single session, non-randomized, balanced trial assessing cognition (58) and a placebo-controlled cross-over treatment case series of three patients (35). Four of these RCTs assessed CBD vs. placebo for antipsychotic augmentation (58, 61–64), while another compared CBD to amisulpride in acutely ill patients off antipsychotics for at least 3 days, which was reported in two papers (59, 60).

A total of 152 stable outpatients and 45 acutely psychotic inpatients with schizophrenia schizophreniform, or brief psychotic disorder were examined. Sample sizes ranged from 15 (63) to 28 (58) in the single dose laboratory studies, and 36 to 88 in the longitudinal clinical trials (59, 61, 62). The CBD studies had heterogeneous study samples and study designs, which are reviewed below.

Regarding demographics, several studies had young adult samples with mean age under 30 (35, 59, 60, 63, 64) and two trials reported mean patient age in the 40s. The majority of participants (58–89%) were male. While the majority of participants identified as White/Caucasian in two studies (61, 62), the other four papers did not provide the race/ethnicity of their samples.

Two studies enrolled outpatients on medication who had chronic illness (58, 61, 62), one enrolled patients on medication who were within 5 years of illness onset (63, 64). Two studies involved chronic patients who were acutely psychotic inpatients at the time of participation (35, 59, 60), and these patients initiated the trial off antipsychotic medication. One study appears to have included a mixed sample of outpatients on or off antipsychotic medications (58).

Three studies excluded participants with cannabis, alcohol, and other substance use or substance use disorders (58–61), but only one used urine drug screens as verification in this process (59). Two studies allowed cannabis use during the trial (62–64). One of these two studies excluded patients meeting criteria for current diagnosis of alcohol or substance dependence or positive drug screen, but allowed current CUD and cannabis use before and during the trial (63, 64). One study did not exclude those with an alcohol or substance use disorder history, and use of all substances was permitted during the trial (62). The case series provided no information pertaining to the inclusion/exclusion of those with a history of alcohol or substance use disorder (35). Nicotine use was generally not excluded, but only one study reported on smoking status (61).

The dose and duration of CBD treatment varied widely across studies. CBD dose ranged from 300 mg to 1,280 mg/day. Three RCTs and the case series provided daily doses over 4 to 6 weeks (35, 59–62). Two administered a single dose (58, 63, 64).

Outcome measures included symptoms, side effects, cognition, ^1^H-MRS, and brain activation as measured using fMRI. Clinical symptoms were most commonly assessed using the Brief Psychiatric Rating Scale [BPRS (65)] and Positive and Negative Symptom Scale [PANSS (66)]. CBD effects on clinical symptoms was reported by five studies (35, 59, 61–63). Side effects were assessed in these same five studies. Motor side effects were commonly assessed using measures such as the Barnes Akathisia Scale [BAS (67)], Simpson Angus Scale [SAS (68)], and the Abnormal Involuntary Movements Scale [AIMS (69)]. Cognition was assessed in five studies (58, 60–63) with a variety of measures, and three included laboratory tests (59, 60, 62, 63). One study evaluated the effects of CBD on a fMRI activation during a verbal learning and memory task (63) and ^1^H-MRS to measure left hippocampal glutamate levels (64). A variety of other measures were occasionally used, such as weight (59, 62) and skin conductance (58).

#### Characteristics of THC Studies

Only three publications report on the effects of THC among patients with schizophrenia (55–57); two examining different data analyses from the same trial (56, 57). These studies included a total of 25 stable, medicated outpatients with chronic schizophrenia, mean age of patients 32.2 (57) and 44.5 (55). The proportion of men ranged from 58.3% (56, 57) to 76.9% (55). Race composition varied, with the proportion identifying as Caucasian ranged from 46% (55) to 100% (56, 57).

One study excluded all substance use disorders except nicotine and caffeine (55), while the other (56, 57) explicitly included CUD. Participants in the D'Souza study (55) were required to abstain from caffeinated beverages, alcohol, and illicit substances from 2 weeks prior to start of testing until study completion, verified via self-report and urine drug screen. In contrast, the Fisher and Whitfield-Gabrieli studies required that patients met criteria for cannabis abuse and/or dependence, and had used the substance within the past month. Patients then abstained from all substances, with contingent reinforcers, except nicotine and caffeine for at least 7 days prior to test sessions, which was verified using the Timeline Follow Back method (70), urine drug screens, and changes in quantitative urine THC to ensure abstinence.

THC dose and route of administration varied in these studies. One used a single dose of 2.5 mg and 5 mg of THC administered intravenously at different sessions (55). Patients in the studies by Fisher et al. and Whitfield-Gabrieli et al. either smoked a single dose of 3.6% THC cigarettes or ingested 15 mg oral THC on one occasion.

All three studies assessed the effects of THC on symptoms using the PANSS, as well as changes in feeling “high” and other symptoms such as “panic” using a Visual Analog Scale (VAS). Fisher et al. and Whitfield-Gabrieli et al. also included formal measures of cannabis withdrawal and craving. The studies included measures of cognition, and two reports used fMRI to assess brain activation during a resting state (56, 57, 63). All of the studies collected blood samples to assess plasma THC, while one also collected cortisol and prolactin (55).

### Study Summaries

#### Effects of THC in Schizophrenia

One double blind RCT assessed the effects of intravenous THC 2.5 and 5 mg vs. placebo in 13 stable, abstinent outpatients with schizophrenia or schizoaffective disorder without any substance use disorder who were stable on antipsychotic medication. Results were compared to 22 healthy participants who had completed a similar protocol (27). Participants received study drug over three sessions, separated by at least 1 week. Abstinence from caffeinated beverages, alcohol, and illicit drugs from 2 weeks was required before testing began until study completion, verified via self-report and urine screens for illicit drugs. Symptoms and cognitive testing was completed 10 and 30 min after infusion, respectively. THC resulted in worsening of positive symptoms (80% of patients had PANNS subscale score worsened by at least 3 points with the 2.5 mg dose). Verbal learning and recall also worsened, and these changes were more prominent for the patient group compared to the healthy participants. Effects on positive symptoms were not different by dose, whereas there was a dose effect on learning and recall. THC also worsened negative symptoms, clinician- and self-related perceptual alterations and movement symptoms (AIMS and akathisia scores). THC increased plasma prolactin and cortisol greater than placebo. The requirement for abstinence from smoking during the testing day could have resulted in nicotine withdrawal-associated exacerbation of symptoms.

Two reports were published from a trial evaluating the effect of oral THC 15 mg or smoked THC from a 3.6% NIDA joint on symptoms, cognition and brain circuitry using fMRI (56, 57). Twelve stable, treated, abstinent outpatients with schizophrenia and CUD were assessed, in contrast to the D'Souza trial, in which CUD was excluded. Alcohol dependence and other illicit substance use disorders were excluded. Patients were abstinent from substances, with the exception of nicotine and caffeine, for at least 7 days prior to MRI scan days, verified via self-report, urine drug screen and quantitative testing thrice weekly. Tobacco smokers smoked a cigarette 90 min prior to scanning. Patients completed two fMRI scan sessions at least 1 week apart. The first (baseline) session was completed without pharmacological manipulation. During the second (drug) session, patients were randomized to either a smoke 3.6% THC cannabis cigarette using an MRI-compatible, hookah-like device immediately prior to scanning (*n* = 6), or ingest a 15 mg THC capsule 3 h prior to scanning (*n* = 6). A group of 12 healthy controls also completed two scanning sessions.

Results from this study were published in two reports. In the first report (56), at baseline, patients showed reduced resting state functional connectivity between the bilateral nucleus accumbens (NAc) seed region and prefrontal cortical regions involved in reward processing (i.e., anterior prefrontal cortex, orbitofrontal cortex, and ventral anterior cingulate cortex), as well as dorsolateral prefrontal and premotor cortices, insula, and parahippocampal gyrus. Only one region, within visual cortex, showed greater connectivity with the NAc in patients than controls. Both smoked and oral THC increased connectivity between the accumbens and prefrontal regions, with greater connectivity associated with higher plasma THC level in the combined patient sample (i.e., smoked cannabis and oral THC). THC was not associated with changes in symptoms or cognition, but scores were not included in the paper. Cannabis craving and withdrawal also did not change with THC vs. placebo in these abstinent participants, but scores were also not reported. Furthermore, no relationship was observed between connectivity and patient ratings of high, liking and craving. The authors interpreted these findings to be consistent with the hypothesis that reward circuitry is disrupted in schizophrenia and CUD, and that by ameliorating this disruption, low dose THC may have the potential to reduce cannabis use in this population.

In further analyses, Whitfield-Gabrieli et al. (57) examined connectivity of the default mode network (DMN) in the 12 patients described above. At baseline, relative to the healthy group, patients showed DMN hyperconnectivity that correlated with greater PANSS positive symptom severity as well as reduced anticorrelation between the DMN and the executive control network (ECN). THC resulted in reduction of this hyperconnectivity and increased DMN-ECN anticorrelation. Furthermore, stronger anticorrelation between DMN and ECN was associated with better performance on a verbal working memory task in the healthy but not the patient group at baseline, and this association emerged in the patient group after THC administration. The authors interpreted their findings to indicate a possible dose effect, with a lower dose of THC providing benefit, improving circuit function, and higher doses of THC potentially disrupting circuits related to psychosis.

#### THC Study Strengths and Weaknesses

Only two controlled trials reported in three papers are available. Both studies have many strengths including use of placebo controls, careful measurement of previous exposure to THC, and a healthy control comparison group as shown in Table 1. Additionally, the D'Souza et al. study (55) utilized two doses of THC, providing a test of dose effect. Only the Whitfield-Gabrieli et al. (57) study reported serum THC levels, confirming moderate increases that corresponded to the study dosing strategy. Both studies had small sample sizes that likely limited their power to detect small effects. The two publications of the fMRI study did not clearly describe the randomization process, a potential for bias, nor report on symptom or cognitive measure scores, thus evidence was indirect, nor did the study report any specific side effects (Tables 1, 2).

**Table 2:** Study quality and assessment of potential for bias.

**Study**	**Random sequence generation**	**Allocation concealment**	**Blinding of participants and personnel**	**Blinding of outcome assessment**	**Complete outcome data / no attrition bias**	**No selective reporting**	**Absence of other sources of bias**	**Directness of evidence**
D'Souza et al. (55)	Unclear	Unclear	Yes	Yes	Yes > 80%	Yes	Yes	High
Fisher et al. (56)	Unclear	Unclear	Unclear^c^	Unclear^c^	Yes > 80%	Yes	No^d^	Medium^f^
Whitfield-Gabrieli et al. (57)	Unclear	Unclear	Yes	Yes	Yes > 80%	Yes	No^d^	Medium^f^
Zuardi et al. (35)	No	No	No	No	Yes > 80%	Yes	No^a^	High
Hallak et al. (58)	No^e^	Unclear	Yes	Yes	Yes > 80%	Yes	Yes	High
Leweke et al. (59)	Yes	Yes	Yes	Yes	Yes > 80%	Yes	Yes	High
Leweke et al. (60)	Yes	Yes	Yes	Yes	Yes > 80%	Yes	Yes	High
Boggs et al. (61)	Unclear	Unclear	Yes	Yes	Yes > 80%	Yes	No^a^	High
McGuire et al. (62)	Yes	Yes	Yes	Yes	Yes > 80%	Yes	No^a^^,^ ^b^	High
O'Neill et al. (63)	Yes	Yes	Yes	Yes	Yes > 80%	Yes	No^a^^,^ ^b^	High
O'Neill et al. (64)	Yes	Yes	Yes	Yes	Yes > 80%	Yes	No^a^^,^ ^b^	High

a *Other source of bias: trial funded or partially funded by pharmaceutical company*.

b *Other source of bias: subjects with cannabis and other substance use were not excluded*.

c *Publication from same parent study (56) indicated there was double blinding*.

d *Principal Investigator received industry funding for other research, but not for this study*.

e *Pseudo-random assignment*.

f *Symptom and cognition scores not reported; fMRI indicators could be considered surrogate outcome*.

#### Effects of CBD in Schizophrenia

In an early placebo-CBD-olanzapine crossover case series, Zuardi et al. (35) evaluated the effects of CBD on symptoms and side effects in three male inpatients with treatment-refractory schizophrenia. Patients first received placebo for 5 days, then CBD on days 6 to 35, titrated from 40 to 1,280 mg/day. On day 36, CBD was replaced by placebo for the next 5 days, and then to olanzapine for 15 days. Symptoms were systematically assessed during each treatment period. In one patient, CBD was associated with a trend toward symptom improvement (BPRS general, positive, and negative symptoms) at the 1,280 mg/day dose, and symptoms worsened following discontinuation. A second patient showed no benefit from CBD, though negative symptoms worsened following discontinuation. The third patient showed “very minimal improvement” of symptoms. Cognition was not assessed. All three patients tolerated CBD well and no side effects were reported.

Hallak et al. (58) examined the effects of CBD 300 or 600 mg vs. placebo on selective attention and electrodermal response in 28 outpatients with schizophrenia using a repeated session, non-randomized design. Participants were assessed with the Stroop Color Word Test to assess selective attention, as well as psychophysiological assessment of skin conductance, given prior research indicating that poorer selective attention is associated with low electrodermal responsiveness in patients with schizophrenia (71). Subjects were assessed in two sessions 1 month apart, with study drug in the second session, in which participants were sorted into three groups matched for age, sex, years of education, and symptom profile. Each group received a single dose of placebo or either 300 or 600 mg CBD and, after 1 h, completed the Stroop and skin conductance assessments. In contrast to hypothesized effects, the 600 mg CBD group made more errors on the Stroop Color Word Test interference condition than the other two groups, reflecting worse selective attention. Furthermore, while the placebo and 300 mg CBD groups improved performance on the second relative to the first session, the 600 mg CBD group did not. Psychiatric symptoms were not reported. This study was limited by very small size and the testing may have been conducted prior to full absorption of the CBD.

Leweke et al. (59, 60) performed a 4 week, double-blind, parallel-group non-inferiority RCT of CBD vs. amisulpride 800 mg in four divided doses among 42 acutely psychotic inpatients with schizophrenia and without substance use disorder. After at least 3 days off antipsychotics, patients received either CBD or amisulpride, titrated over 1 week, and maintained at 800 mg for an additional 3 weeks. Symptoms (positive, negative, and total), reported in the 2012 report, improved in both groups, including a 30-point reduction in PANSS total symptom scores and about a nine-point reduction in positive symptoms by the 4 week endpoint. There was no group difference in symptom improvement, suggesting that CBD had an antipsychotic effect similar to amisulpride, although the non-inferiority test did not achieve significance (59). Results from a battery of cognitive tests administered pre- and post-treatment, reported in the 2021 report, demonstrated that both groups showed improvement of visual memory and processing speed. The CBD group only improved in sustained attention and visuomotor coordination, while the amisulpride group improved in working memory. These cognitive findings, however, were not statistically significant after correction for multiple comparisons (60). CBD was well-tolerated and associated with fewer extrapyramidal symptoms, less weight gain, and lower prolactin increase than amisulpride (60). Furthermore, serum anandamide levels increased more among those treated with CBD than amisulpride, and the extent of increase was associated improvement in PANSS total score in the CBD group but not the amisulpride group. This finding was interpreted as suggesting a link between the antipsychotic effect of CBD and inhibition of anandamide degradation (72). Anandamide levels and PANSS scores were not correlated with cognitive performance. The authors interpreted this finding as suggesting a different mechanism for the effect of CBD on cognition. Treatment groups were small and the study was underpowered due to enrollment challenges.

Two 6 week, placebo-controlled trials assessed the efficacy of CBD augmentation of antipsychotics. In the first, Boggs and colleagues (61) conducted a 6 week, double blind, parallel group RCT of CBD 300 mg BID vs. placebo among 36 outpatients with chronic schizophrenia and no past 3 month substance use disorder on a stable dose of antipsychotic medication. Mean age was 48. Psychotic symptoms decreased over time, but improvement was not different between treatment conditions (PANSS positive symptom scores improved 2–3 points). In contrast to the direction of the hypothesized effect, the placebo group showed small improvements in MCCB Composite score, as well as Reasoning and Problem Solving domain scores. Sedation was greater (20% vs. 5%), and gastrointestinal symptoms were less frequent (33.3% vs 55.5%) in the CBD group.

In the second 6 week augmentation trial, McGuire and colleagues (62) conducted a 6 week, double-blind, parallel-group RCT of a higher dose of CBD (500 mg BID) vs. placebo among a larger group, 88 patients with schizophrenia spectrum disorders, mean age 41 years, who were stable with at least a partial response to antipsychotic treatment. In addition to using a higher dose of CBD, this trial differed from the previous trial in that substance use disorder was not exclusionary and use of alcohol, cannabis, or other illicit substances was not prohibited during the trial, but DSM-5 substance use disorder diagnosis was not reported. At baseline, only 2.3% of the CBD group and 4.4% of the placebo group had a THC-positive urine screen, suggesting that most participants were not regular cannabis users prior to the study. Serum CBD levels were positive in all participants in the CBD group at study end suggesting adequate adherence. Following the 6 week treatment phase, compared to placebo, the CBD group showed greater improvement in positive symptoms (3.2 vs. 1.7 point reduction) and was more likely to be rated as improved by their treating clinician. Total PANSS score change was not significantly different between groups (7.9 vs. 8.9 points). Trends were also observed for cognition (composite score and executive function domain), as well as small but significant amelioration of motor speed. Although information on substance use during the course of the study was generally not provided, the authors reported that one patient in the CBD group was cannabis dependent at baseline and did not change their pattern of use during the study, and another in the CBD group was alcohol dependent at baseline, but not by the end of treatment. CBD was well-tolerated, but, in contrast to the Boggs study above, CBD participants did not report somnolence (0% vs. 6.7%), and were more likely to have gastrointestinal side effects than the placebo group (18.6% vs. 6.7%). Because participants using THC were not excluded and measures of THC use were not systematically assessed over time, an interaction between use of cannabis or other substances during the study and the effect of CBD on symptoms could not be ruled out.

O'Neill et al. (63, 64) evaluated the effect of a single dose of augmentation with CBD 600 mg vs. placebo on symptoms, fMRI assessments of mediotemporal and prefrontal cortex (primarily the middle frontal and inferior frontal gyri) activation, as well as mediotemporal–striatal functional connectivity during verbal recall, and ^1^H-MRS assessment of hippocampal glutamate level (which was corrected for the cerebral spinal fluid content of the hippocampal region of interest). They studied 13 medicated outpatients with schizophrenia (within 5 years of illness onset; mean age 28), in a double blind, repeated-measures, within-subject cross-over design. Patients with CUD were allowed whereas alcohol and other substance dependence were excluded, as were those who were intoxicated or had a positive urine drug screen for other drugs on the day of scanning. Over half (57.1%) of patients were using cannabis. Nineteen healthy comparison (HC) participants also completed two sessions, but without drug administration for the fMRI study. All participants completed a block-design verbal paired associate learning task (engaging learning and memory) in the scanner 3 h after drug administration.

CBD was associated with a trend toward reduced median PANSS total score, but not with changes in state anxiety or verbal paired associate learning task performance. As compared to the healthy group, patients had abnormal activation within the prefrontal region during encoding, while during recall they had abnormal prefrontal and mediotemporal activation as well as greater hippocampal-striatal functional connectivity. CBD partially normalized activations in these regions, as well as reduced hippocampal-striatal functional hyperconnectivity. The researchers interpreted their findings to indicate that the changes in these regions underlie the antipsychotic effects of CBD.

Furthermore, in a follow-up report, O'Neill et al. (64) observed a significant increase in left hippocampal glutamate levels in the CBD group compared to placebo. No group differences were observed for other metabolite levels including glutamate–glutamine, myoinositol, N-acetyl aspartate, and glycerophosphocholine. A multivariable model adjusted for baseline PANSS score demonstrated a significant inverse predictive relationship between glutamate levels, but not CBD condition, and total PANSS scores. The authors interpreted these findings to be supportive of the possibility that CBD may produce an antipsychotic effect via modulation of hippocampal glutamate levels.

The study sample was small but the authors provided a power calculation indicating adequate power for the fMRI study. The design included adequate time for CBD absorption, enabling detection of drug effect. However, because half of participants were using recreational cannabis, the authors could not determine whether the CBD-associated improvements were due to ameliorating THC-induced impairments vs. impairments fundamental to schizophrenia.

#### CBD Study Strengths and Weaknesses

All five studies had considerable strengths with prospective random assignment, a control or comparison condition, and systematic assessment of symptoms and/or cognition. The 4 and 6 week trials also carefully measured impact on movement disorders and adverse effects. In the single dose trials, one study may not have included adequate time for absorption of oral CBD. The different sample characteristics (age, presence of CUD, or recent use of cannabis), different CBD dose, treatment duration, outcome measures and timing of assessments could contribute to the heterogeneity of findings. A notable point of study design heterogeneity is the inclusion or exclusion of CUD and/or cannabis use during the trial; both studies with positive findings did not omit participants with CUD. The small sample sizes of these studies limited the power to detect small to medium effects. The Boggs study did not clearly describe the randomization process and pharmaceutical company funding for some of these studies could contribute some potential for bias in the findings (Table 2).

## Discussion

### THC, Psychotic Symptoms, Cognition, and Adverse Effects

Controlled laboratory research to date has used heterogonous methodology and reported different findings. The D'Souza study, which was carefully designed to assess symptoms, documented increased positive, negative, and general symptoms of psychosis, as well as impaired cognition when intravenous THC was given to patients with schizophrenia. While there was a clear dose effect for learning and recall, there was not a clear dose effect for positive symptoms (55). These results are consistent with findings in healthy subjects, where 15 trials have demonstrated that THC can induce psychosis in many people (28).

In contrast, the other study, which included patients with schizophrenia having co-occurring CUD, did not report symptom changes with administration of a modest dose of oral and smoked THC; THC significantly increased serum THC and resulted in a trend toward tachycardia, as expected. This study demonstrated that THC reduced the resting state functional hyperconnectivity in regions of the DMN and improved the DMN-ECN anticorrelation in brain circuits associated with schizophrenia symptomatology (57), an effect that is opposite of what might be expected if THC worsened psychosis.

Hyperconnectivity of the DMN has been reported in medicated (73) and medication naïve patients with schizophrenia (74) who do not have CUD. The decreased DMN-ECN anticorrelation found has also been documented in medication naïve (74–76) and chronic patients taking medication (73, 77, 78). Thus, the authors asserted that these abnormalities may be core features of schizophrenia. They interpreted their findings to indicate that THC may have a dose effect, with low dose providing benefit to brain circuits involved in psychosis, and higher doses causing disruption. The other report from this study showed a normalization of resting state activity in circuits involved with reward (56), and proposed that low dose THC could also have the therapeutic potential to reduce cannabis use in patients with co-occurring schizophrenia and CUD.

Regarding the effect of THC on cognition in schizophrenia, intravenous THC worsened learning and recall in patients with schizophrenia without substance use disorder, with a dose effect in which 5 mg had a greater effect than 2.5 mg (55). Although the Whitfield-Gabrielli study in abstinent patients with CUD reported that THC improved anticorrelation between the DMN and ECN, and the magnitude of the anticorrelation between the DMN and ECN correlated with working memory performance, cognition scores in relation to THC vs. placebo were not reported. The THC effect in the D'Souza study is consistent with findings that THC acutely worsens cognition in the general population (55) as well as a meta-analysis indicating better neuropsychological functioning in patients with schizophrenia having a lifetime history of cannabis use, but not those with current or recent use, relative to patients without co-occurring cannabis use (79).

In addition to the different dose effect suggested by Whitfield-Gabrielli et al. (57), other potential explanations for the different symptom and cognition findings regarding THC and psychotic symptoms in these studies of chronic schizophrenia include the possibility that patients with schizophrenia and co-occurring CUD may be less susceptible to the psychotomimetic effects of THC than those who do not have a CUD, either due to different underlying biological risk, a notion that others have proposed (80), or due to developing neural adaptations resulting in tolerance to this effect after long term cannabis use. Regarding heterogeneous biological risk for psychosis, inter-individual susceptibility to THC-induced psychotic symptoms has been observed in people without psychotic disorders (27, 81). Assuming such heterogeneity also exists in people with schizophrenia, it is possible that those with lower susceptibility to symptom exacerbation may be more likely to develop a CUD, as they would not suffer immediate negative consequences with using THC. The problematic course of illness associated with CUD in schizophrenia may be due to the more general impairing impact of substance use disorders in schizophrenia, including medication non-adherence (82–84). Alternatively, people with schizophrenia and CUD may have developed tolerance to the psychotogenic effects of THC, as has been demonstrated in people without psychotic disorders (81, 85).

### CBD, Psychotic Symptoms, Cognition, and Side Effects

Controlled prospective research on the impact of CBD to date is mixed. The small study comparing 800 mg CBD to amisulpride among 42 symptomatic, unmedicated inpatients (mean age 30 years) who tested negative for THC and substance use disorder demonstrated a 30 point reduction in PANSS total scores over 4 weeks and about a 9 point reduction in positive symptoms of psychosis in both groups (59). Although this study did not have a placebo control group, the findings strongly suggested that CBD has an antipsychotic effect. A recent paper also reported on assessments of cognition from this same study, indicating similar levels of improvement with CBD and amisulpride, but without statistical significance after correction for multiple comparisons (60). The four small placebo controlled studies of CBD augmentation in schizophrenia provide mixed, limited support for the ability of CBD added to an antipsychotic to further reduce symptoms of psychosis and improve cognitive impairments. In contrast to the research on THC, this research did demonstrate that CBD did not worsen psychosis or cognition compared to placebo.

These inconsistent results regarding potential beneficial effects of CBD could be due to differing doses of CBD, differing patient age, and presence of recent/current recreational THC and other substance exposures in these studies. Among the two 6 week augmentation trials, the study that demonstrated a positive effect on symptoms and cognition (62) used a higher dose of CBD (1,000 mg vs. 600 mg) and enrolled subjects with a lower mean participant age (41 vs. 48 years). Thus, it is possible that a higher dose is necessary, or that younger patients may respond better to CBD. The findings of the effect of CBD using fMRI in the studies reviewed here (63), which also recruited young subjects, are similar to recent fMRI studies in young, antipsychotic-naïve adults at clinical high risk for psychosis. These trials found partial normalization of circuitry involved in verbal learning and memory (86) and motivational salience (87) following a single dose of CBD. The novel ^1^H-MRS findings suggest a possible mechanism for the impact of CBD on symptoms in schizophrenia (64). Together, these findings suggest that the effects of CBD on brain functioning in schizophrenia cannot be readily accounted for by illness-related factors such as medication history and chronicity.

A point of significant interest is that studies finding a positive effect for CBD augmentation (62–64) did not omit participants with CUD or current cannabis use and did not carefully measure cannabis use throughout the study period. Previous research has demonstrated that CBD in robust doses can mitigate THC-induced psychotic symptoms in healthy individuals (29, 30). Thus, it is possible that CBD was influencing THC-induced impairments rather than impairments due to schizophrenia in these two studies with positive results. It is also possible that people with schizophrenia who use cannabis or have a CUD may respond differently to CBD that those who do not have CUD, but no studies have carefully examined the effect of CBD in patients with schizophrenia and CUD. Additionally, we found no published laboratory studies testing the combination of CBD with THC in schizophrenia, nor among those with co-occurring CUD. Patients with co-occurring disorders are of particular interest given the preliminary findings that low dose THC normalized resting state functional connectivity in areas related to reward processing and executive control without increasing symptoms or worsening cognition (56, 57).

This review is limited by the small number of controlled studies available on the topic, yet the consideration of studies of both CBD and THC together with careful review of study methodology and findings provides an important current appraisal of the evidence on the effect of cannabis in schizophrenia. Importantly, prior reviews have not taken into careful consideration whether patients were using alcohol or substances of abuse (including cannabis) at the time of participation and/or had a prior history of alcohol or substance use disorder. Alcohol/substance use history may be especially salient to consider as it may affect the outcomes of THC or CBD trials in schizophrenia. This possibility is raised by research indicating differential effects of acute cannabinoid administration on cognition (88, 89) and ratings of intoxication (90, 91) in frequent and infrequent cannabis users without schizophrenia, as well as higher initial maximal plasma THC level in frequent users (90, 92).

Overall, there is insufficient evidence regarding the ability of THC or CBD to impact symptoms and cognition in patients with schizophrenia, such that neither cannabinoid should be recommended for treating this group until further research enables a clearer picture of their impact on this disease and among people who have schizophrenia and CUD. In the era of legalization, public health officials could consider whether there is enough THC-related evidence (from one high quality laboratory study that is consistent with epidemiologic research and effects in people without psychotic disorders) to provide public warnings that THC can worsen symptoms among some people with schizophrenia. Studying the effect of THC and CBD in schizophrenia is challenging, but additional research is warranted to examine the impact of these cannabinoids among individuals with schizophrenia who do and do not have co-occurring CUD.

## Data Availability Statement

The original contributions presented in the study are included in the article/supplementary material, further inquiries can be directed to the corresponding author/s.

## Author Contributions

CS and MB determined study design and contributed to developing the original protocol. SA and CS contributed to the original screening of papers, data extraction, and writing the first draft of the manuscript. RR contributed to the study design. MB and RR conducted analysis, interpretation of literature review, and critical revision of the manuscript. MB, RR, and SA contributed to editing the final draft. All authors approved the final version.

## Conflict of Interest

The authors declare that the research was conducted in the absence of any commercial or financial relationships that could be construed as a potential conflict of interest.

## Publisher's Note

All claims expressed in this article are solely those of the authors and do not necessarily represent those of their affiliated organizations, or those of the publisher, the editors and the reviewers. Any product that may be evaluated in this article, or claim that may be made by its manufacturer, is not guaranteed or endorsed by the publisher.
